# Bambara Nut Root-Nodules Bacteria from a Semi-Arid Region of South Africa and Their Plant Growth-Promoting Traits

**DOI:** 10.1155/2023/8218721

**Published:** 2023-06-30

**Authors:** Ayansina Segun Ayangbenro, Mohomud Rashid Adem, Olubukola Oluranti Babalola

**Affiliations:** Food Security and Safety Focus Area, Faculty of Natural and Agricultural Sciences, North-West University, Private Bag X2046, Mmabatho 2735, South Africa

## Abstract

Rhizobial nitrogen-fixing bacteria are the main inhabitants of the root nodules of legume plants. Studying the bacterial community of legume nodules is important in understanding plant growth and nutrient requirements. Culture-based technique was used to examine the bacterial community of these underground organs from *Vigna subterranea* L. Verdc (Bambara nut), an underutilized legume in Africa, for plant growth-promoting traits. In this study, Bambara nuts were planted to trap root-nodule bacteria, and the bacteria were morphologically, biochemically, and molecularly characterized. Five selected isolates were screened *in vitro* for their plant growth-promoting traits and exhibited differences in their phenotypic traits. The polymerase chain reaction (PCR) products were subjected to partial 16S rRNA gene sequencing for phylogenetic analysis. Based on 16S rRNA gene sequence, the isolates were identified as BA1 (*Stenotrophomonas maltophilia*), BA2 (*Chryseobacterium* sp.), BA3 (*Pseudomonas alcaligenes*), BA4 (*Pseudomonas plecoglossicida*), and BA5 (*Pseudomonas hibiscicola*). Results showed that four of the five isolates could produce IAA. The capability to solubilize phosphate in Pikovskaya's agar plates was positively shown by four isolates (BA2, BA3, BA4, and BA5). Three isolates could produce hydrogen cyanide while isolates BA1, BA3, BA4, and BA5 were found to have ammonia-production traits. The results suggest that these plant growth-promoting isolates can be used as inoculants for plant growth and productivity.

## 1. Introduction

Bambara nuts remain one of the neglected and underutilized crops in Africa, where smallholder farmers primarily grow them. Culture and traditional practices maintain these crops, but research and conservation remain inadequately characterized and neglected. Their potential value has been underestimated and underutilized due to a lack of attention [1]. Underutilized and neglected crop species are nutritionally rich; therefore, their erosion can negatively affect the nutritional status and food security of the poor. Their enhanced use can help to fight hidden hunger and improve nutrition [1].

The rural poor rely on Bambara nut for food security, nutrition, and income generation. With Africa's population on the increase, there is a need to ensure affordable foods. *Vigna subterranea* L. Verdc. (Bambara groundnut) is the third most important food legume after *Arachis hypogea* (groundnut) and *Vigna unguiculata* (cowpea) in Africa [2]. Legumes, such as Bambara nuts, play a major role in the fight against malnutrition [3]. Bambara nuts possess high crude protein content between 22 and 37% [4, 5]. Due to its high lysine and methionine content, it provides a significant supply of protein, particularly for rural and urban residents who cannot afford the expensive price of animal protein. The potential of neglected and underutilized crops such as Bambara nuts might be used to address the continent's food shortages [6]. Therefore, it is essential to increase Bambara nut consumption and knowledge levels, which are currently too low in many developing countries.

Both plants and microorganisms have evolved to take advantage of their close relationship. The plant microbiome determines plant health and productivity. The phytomicrobiome contains a wide variety of bacterial and fungal microorganisms that colonize all parts of the plant, particularly the roots [7]. These organisms transform essential nutrients into useable forms that can be assimilated by plants while plants release a variety of chemical substances into the rhizosphere, called exudates, which mediate the interaction between plants and microbes [8, 9]. Pathogen resistance, water retention, and the production of growth-stimulating hormones are benefits conferred on plants by beneficial phytomicrobiome. The microbial community of plants differs across plant genotypes, plant organs, plant growth stages, and locations [9].

The recent advancements in undersatanding the mechanisms emplyoyed by phytomicrobiome in stimulating plant growth and development are expected to continue to increase [10]. A paraphyletic genus of bacteria known as rhizobia within plant tissues transforms atmospheric nitrogen to ammonia by the highly specialized microbial community found in the root nodules of leguminous plants [7]. Rhizobia differ in how they approach nodulation. While some of them can only nodulate certain plant species, including nonlegume plants, others can nodulate various plant species. Rhizobia are a group of genetically varied microorganisms. They cooperate to fix atmospheric nitrogen in a process known as symbiotic nitrogen fixation [11]. In addition to rhizobia that fix nitrogen, there are different types of nonrhizobial bacteria found in this ecological niche that engage in a variety of biological activities, such as biocontrol and stimulation of plant development [11]. Finding bacterial strains with strong rhizobium-legume associations to increase agricultural productivity requires the isolation and characterization of rhizobia, which is a rich biological resource. Rhizobial bacteria increase plant productivity and growth by generating a variety of compounds. Therefore, a complete understanding of the biology of bacteria in legume nodules is critical.

Bambara nut is the perfect crop for farmers, especially on marginal soils because it can generate high yield levels with little input. The production of Bambara nuts in South Africa has been reported in various literatures [8, 12, 13]. There is an increase in the study of plant growth-promoting bacteria (PGPB) globally, especially in sustainable agriculture, since they can reduce the use of chemical fertilizers [14]. Basic and applied research is being conducted to develop efficient bacterial strains that may be employed as plant growth promoters to attain targeted crop output and yield due to its advantageous impacts on agriculture. The aim of this study was to identify and characterize bacterial populations from root nodules of Bambara nuts and their *in vitro* plant growth-promoting traits.

## 2. Materials and Methods

### 2.1. Sampling Site and Sample Collection

The root nodules of the Bambara nut used in this study were obtained from the North-West University Agricultural Field, Mahikeng, which is located at latitude S 25°49′35.1559″ and longitude E 25° 36′39.2826″. Twenty healthy plants, without the manifestation of any feasible disease, were selected for the study. After carefully removing the soil surrounding the roots so as not to harm them, the roots themselves were pulled out. In sterile zip-lock bags, the roots were then transported to the laboratory and kept at −20°C.

### 2.2. Bacterial Isolation

The root nodules were shaken to remove the attached soils, and the nodules were thoroughly washed under tap water. The nodules were then surface sterilized by soaking in 70% ethanol for 10 s before being repeatedly washed with sterile distilled water. The effectiveness of the surface sterilization procedure was tested by rolling the surface-sterilized nodules over nutrient agar (NA) before crushing the nodules to isolate rhizobia. Similarly, an aliquot of 100 *μ*l of water from the last rinse phase was plated on NA plates and tested for the growth of contaminants.

With the aid of a sterile pestle and mortar, the nodules were crushed into a paste after the removal of water droplets from the surface. Rhizobia were isolated from the root nodules that had been properly surface sterilized. Exudates from the crushed nodules were plated on NA plates and incubated at 28°C for 48 h. The visible colonies were subcultured on fresh NA and incubated at 28°C for 24 h to obtain pure cultures.

### 2.3. Morphological and Biochemical Characterization of Isolates

The morphological and biochemical tests were performed for the characterization of the isolates using standard methods and a miniaturized multitest identification system API 20E and API 20NE test kit (BioMérieux) according to the manufacturer's instructions. API 20E and API 20NE are standardized biochemical test for the identification and differentiation of members of the family Enterobacteriaceae and nonfastidious, nonenteric Gram-negative rods (for example, *Acinetobacter*, *Aeromonas Pseudomonas*), respectively.

### 2.4. *In Vitro* Screening of Isolates for Plant Growth-Promoting Traits

#### 2.4.1. Production of Ammonia

Bacterial isolates were tested for ammonia production using peptone water. Fresh culture of bacterial isolates was inoculated into peptone water (10 ml) in a tube and incubated at 28°C for 48 h. After that, 0.5 ml of Nessler's reagent was added to the tube. A positive ammonia production test result was the transformation of brown to yellow [15].

#### 2.4.2. Hydrogen Cyanide (HCN) Production

Fresh bacterial cultures were screened for HCN production as described by Lorck [16]. Isolates were grown in nutrient broth amended with 4.4 g·L^−1^ of glycine and streaked on nutrient agar plates. The top of the plate was covered with sterile Whatman filter paper no. 1 which had been dipped in a solution of 2% sodium carbonate and 0.5% picric acid. Parafilm was used to seal the plates, which were then incubated at 28°C for 4 days. HCN generation was identified by the yellow color on the Whatman filter paper turning dark brown.

#### 2.4.3. Indole Acetic Acid (IAA) Production

The Bric et al. [17] protocol was used to measure IAA production by the isolates. Bacterial cultures (100 *μ*l) were added to nutrient broth (5 ml) supplemented with 0.1% tryptophan, and the mixture was incubated at 28°C for 48 h. After incubation, the cells were harvested by centrifugation at 10,000 rpm for 10 minutes. Then, the Salkowski reagent (50 ml, 35% perchloric acid, and 1 ml of 0.5 M FeCl_3_ solution) was combined with 2 ml of the supernatant, 2 drops of 10 mM orthophosphoric acid, and 4 ml of the Salkowski reagent. For 25 minutes, the mixture was incubated at room temperature. Pink color development suggested IAA production.

#### 2.4.4. Phosphate Solubilization

By spotting 10 *μ*l of each bacterial isolate on Pikovskaya's agar and incubating it at 28°C for seven days as reported by Nautiyal [18], the ability of the isolates to solubilize phosphate was determined. The ability of the isolate to solubilize phosphate was shown by the presence of a distinct halo zone surrounding the culture spot.

### 2.5. Molecular Characterization of the Bacterial Isolates

The extraction of genomic DNA was carried out using the ZR soil microbe DNA (Zymo Research) kit as described in the kit protocol. The 16S rRNA gene nucleotide sequences were determined by PCR using a C1000 thermal cycler (BioRad, CA, USA). The fD1 (5′-AGAGTTTGATCCTGGCTCAG-3′) and rP2 (5′-ACGGCTACCTTGTTACGACTT-3′) primers were used for amplification [19]. A final volume of 25 *μ*l was used for the PCR reactions, which contained 12.5 *μ*l of 2× master mix (One Taq® Hot Start Quick Load, Biolabs, England), 0.5 *μ*l of each primer, and 1 *μ*l DNA. The thermal cycling settings were initial denaturation for 5 min at 94°C, followed by 30 cycles of 30 s at 94°C, annealing for 1 min at 54°C, and extension for 2 min at 72°C. The PCR result was sequenced using an ABI PRISM® 3500XL DNA Sequencer (Applied Biosystems) at Inqaba Biotechnical Industrial (Pty) Ltd in Pretoria, South Africa. The sequences were analyzed and edited using Bio Edit [20]. Evolutionary distance matrices were generated according to Jukes and Cantor [21]. Phylogenetic and molecular evolutionary analyses were performed with MEGA version 5.2.2 [22], and a phylogenetic tree was constructed using the neighbor-joining method [23]. Bootstrap analysis was performed using 1000 replications for the neighbor-joining data set.

## 3. Results and Discussion

### 3.1. Isolation and Characterization of Bacterial Isolates

Five bacterial isolates were isolated from the nodules of Bambara nuts and further characterized. All of the bacterial isolates were Gram-negative and rod-shaped, as shown in Table 1. The pigmentation of isolates varies from cream, orange, and yellow to white. All the isolates were catalase positive, while only 60% were oxidase positive. Further biochemical tests were performed with API 20E test kit, which showed that the five isolates were positive for citrate utilization, hydrolysis of gelatin, and acetone production (Table 2). All isolates were negative for indole and H_2_S production. None of the isolates could use sucrose, mannitol, inositol, or sorbitol as the carbon source. As stated by Etesami [24], rhizobia are Gram-negative bacteria, which can induce nodules on legumes. The ability of these organisms to transform atmospheric nitrogen for their leguminous hosts in exchange for carbon is well documented. Members of the class alphaproteobacteria and the genera Rhizobium, Ensifer, Phyllobacterium, Allorhizobium, Bradyrhizobium, Devosia, Azorhizobium, and Microvirga transform atmospheric nitrogen into forms usable by plants in the nodules of legumes.

Other bacterial species (nodule-associated bacteria) exist in the nodules of legumes in addition to rhizobial, which are involved in different biological activities. Beijerinck [25] provided the earliest account of isolating *Bacillus* species from root nodules. Afterwards, research based on strain isolation revealed a wide variety of bacteria—outside of rhizobia—associated with root nodules. *Acinetobacter, Bacillus, Agrobacterium, Micromonospora, Enterobacter, Mycobacterium, Pantoea,* and *Pseudomonas* species make up the majority of the nonrhizobial bacteria in the ecological niche [26–28]. The findings showed that nonrhizobial bacteria are widely distributed and abundant in the nodules of Bambara nuts. It has been suggested that the root nodules of legumes may develop a niche that will allow nonrhizobial bacteria to survive and thrive [29]. When rhizobial bacteria are present and infected, these nonrhizobial strains can infiltrate the root nodules of legumes [24], which may account for the nonrhizobial bacteria isolated in this study. In crop production, the resident bacteria in the root nodules are essential for the growth, and survival of legumes and their interactions inside nodules will have an impact on plant health.

### 3.2. Plant Growth-Promoting Traits Screening

Bacterial strains were screened for various plant growth-promoting traits, such as ammonia, HCN, IAA production, and phosphate solubilization (Table 3). Isolates BA 3, 4, and 5 were positive for all the plant growth-promoting tests, while BA1 tested positive for ammonia and IAA production and BA2 positive for only phosphate solubilization. Despite not being able to fix atmospheric nitrogen or produce nodulation, several nonrhizobial bacteria present in the root nodules of legumes that are not members of the phylum. Proteobacteria have been found to possess many plant growth-promoting features [24]. Hydrogen cyanide, IAA, siderophore, and 1-aminocyclopropane-1-carboxylate deaminase production, phosphate solubilization, and the capacity to inhibit pathogenic fungi are a few of these traits [30, 31].

Due to its significant toxicity against phytopathogens, HCN has been extensively used in agriculture as a biocontrol agent. It can also be used in chelating metal ions and indirectly helping to make phosphate available to plants [32]. It is a crucial biocontrol characteristic for protecting crops. It is a phytotoxic substance that prevents key metabolic enzymes from functioning [33]. Consequently, there is a growing interest in their use as biocontrol agents. *Pseudomonas* frequently produces HCN [34]. The ability of PGPB to produce HCN, which is crucial for the biological control of many soilborne pathogenic fungi, is another key characteristic [35]. To create efficient inoculants for crop production, it is critical to characterize the distribution of rhizobia and screen for PGR traits. Hydrogen cyanide production by bacterial isolates in this study could increase nutrient availability for plants, promote plant growth, and decrease the incidence of pathogen attack.

Plant growth-promoting bacteria are natural potential resources that colonize roots and are directly or indirectly involved in promoting plant growth [36]. The use of PGPB for legume and nonlegume crops has been considered since they have the potential for growth stimulation [37]. *Bradyrhizobium* strains coated on peanut seeds considerably improved seed germination, biomass, nodule number and fresh weight, and average nodule weight according to Deshwal et al. [38].

The phytohormone IAA is recognized for promoting plant growth by improving seedling growth, cell differentiation, and root elongation [39, 40]. Several root-nodule bacteria have been reported to produce IAA [41–43]. The building block for IAA synthesis is tryptophan, and its availability increases IAA production [44]. Indole-3-acetic acid is important in shaping root architecture, such as the differentiation of vascular tissue, regulation of lateral root initiation, gravitropism, and meristem maintenance [45, 46]. In this study, four isolates *Stenotrophomonas maltophilia*, *Pseudomonas alcaligenes*, *P. plecoglossicida* and *P. hibiscicola* were found to produce IAA.

Phosphorus is an essential nutrient in the growth of plants [47]. A class of helpful bacteria known as phosphate-solubilizing bacteria can hydrolyze organic and inorganic phosphorus from insoluble substances. Solubilization of phosphate is an important mechanism of plant growth promotion. Biogenic phosphorus is made available to plants through phosphatase secretion to release phosphorus bound in organic matter and the formation of organic acids and chelating compounds that aid in lowering rhizosphere pH [48, 49]. Several phosphate-solubilizing bacteria have been documented from the root nodules of legumes [50–52]. Four of the five isolated bacterial species from this study could solubilize inorganic phosphate. This study showed that the isolated bacteria can solubilize phosphate, and these bacteria as proposed by Ekin [53] are crucial for making the solubilized fraction of different phosphate minerals in soil accessible to plants for growth.

Single strains of rhizobacteria can exhibit multiple PGP traits, based on screening for PGP-associated compounds [39, 54]. Nevertheless, their effects are unique, and no two systems that differ in the kind of soil, the plant genotype, or the combination of rhizobacteria in the community will demonstrate the same plant growth promotion [55]. A single bacterial strain will not have the same beneficial effect in two distinct conditions, and strains that do not exhibit any PGP traits *in vitro* may nevertheless be able to stimulate plant growth or vice versa [56]. Multitrait plant growth-promoting bacteria are organisms that support plant growth through a variety of mechanisms, and these traits increase seedling emergence, vigor, and yield [57]. The bacterial isolates from this study have multiple plant growth-promoting properties that can be harnessed for plant growth in marginal soils.

### 3.3. Molecular Characterization of Isolates

**Figure 1:**
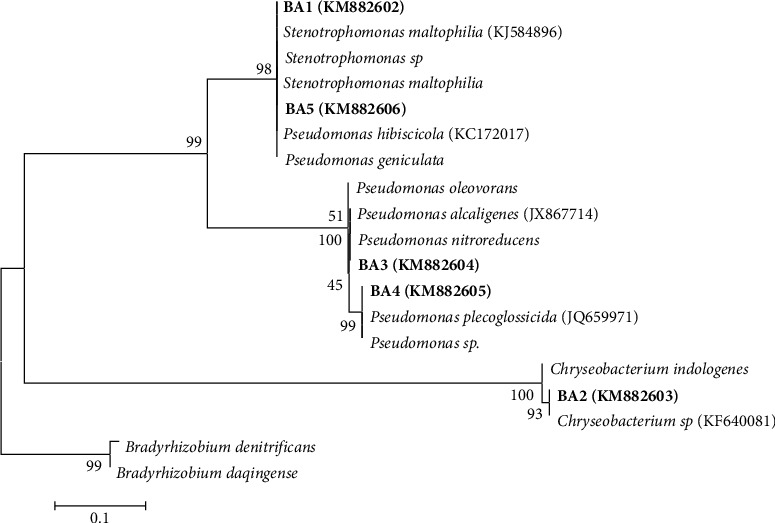
Neighbor-joining phylogenetic tree from 16S rRNA gene sequences of bacterial isolates.

To confirm the strain identification of each bacterial isolate, a polymerase chain reaction was used to conduct partial 16S rRNA gene sequencing. For accession numbers, the sequences were deposited in GenBank. Results from the GenBank nucleotide sequence analysis using BLAST are presented in Table 4. Based on incomplete 16S rRNA gene sequences, Figure 1 shows the neighbor-joining tree of typical species and bacterial isolates. Root-nodule bacteria of various genera, such as *Stenotrophomonas, Chryseobacterium,* and *Pseudomonas*, symbiotically coexist with Bambara nuts. This was demonstrated by comparing our 16S rRNA gene nucleotide sequences with those published in the NCBI gene bank database (Table 4).

## 4. Conclusion

The results of this study illustrate that the bacterial isolates from the root nodules of Bambara nuts have various PGPR activities. These isolates may be suitable for use as inoculants to promote plant development. The mechanisms that contribute to growth promotion need to be better understood. Further research in PGPR field applications would aid in that role and improve agricultural production, thus helping solve malnutrition and the attainment of food security.

## Figures and Tables

**Table 1:** Morphological and biochemical characterizations of bacterial isolates.

Isolates	Gram staining	Cell shape	Pigmentation	Oxidase test	Catalase test
BA1	−	Rod	Cream	−	+
BA2	−	Rod	Orange	+	+
BA3	−	Rod	Yellow	−	+
BA4	−	Rod	Cream	+	+
BA5	−	Rod	White	+	+

**Table 2:** API 20E-based biochemical characterization of the bacterial isolates.

Tests	Reaction	Negative	Positive	BA1	BA2	BA3	BA4	BA5
ONPG	*β*-Galactosidase	Colourless	Yellow	+	−	+	−	−
ADH	Arginine dihydrolase	Yellow	Red or orange	+	+	+	+	+
LDC	Lysine decarboxylase	Yellow	Red or orange	+	−	−	−	−
ODC	Ornithine decarboxylase	Yellow	Red or orange	+	−	−	−	−
CIT	Citrate utilization	Pale green or yellow	Blue-green or blue	+	−	−	+	+
H_2_S	H_2_S production	Colourless or grey	Black deposit	−	−	−	−	−
URE	Urea hydrolysis	Yellow	Red or orange	+	−	−	−	−
TDA	Tryptophan deamination	Yellow	Reddish brown	+	+	+	+	+
IND	Indole production	Colourless pale green or yellow	Pink	−	−	−	−	−
VP	Acetoin production	Colourless	Pink or red	+	+	+	+	+
GEL	Gelatin hydrolysis	No diffusion	Diffusion of black pigment	+	+	+	−	−
GLU	Glucose fermentation	Blue or blue-green	Yellow or greyish yellow	+	+	+	+	+
MAN	Mannitol	Blue or blue-green	Yellow	−	−	−	−	−
INO	Inositol	Blue or blue-green	Yellow	−	−	−	−	−
SOR	Sorbitol	Blue or blue-green	Yellow	−	−	−	−	−
RHA	Rhamnose	Blue or blue-green	Yellow	−	−	−	+	+
SAC	Sucrose	Blue or blue-green	Yellow	−	−	−	−	−
MEL	Melibiose	Blue or blue-green	Yellow	+	−	−	+	+
AMY	Amygdalin	Blue or blue-green	Yellow	+	−	−	—	—
ARA	Arabinose	Blue or blue-green	Yellow	+	−	−	+	+

**Table 3:** Characteristics of plant growth-promoting rhizobacteria isolated.

Isolate	Ammonia production	HCN production	IAA production	Phosphate solubilization
BA1	+	−	+	−
BA2	−	−	−	+
BA3	+	+	+	+
BA4	+	+	+	+
BA5	+	+	+	+

**Table 4:** Results of the BLASTN algorithm's comparison of the 16S rRNA gene sequences of bacterial isolates and GenBank accession numbers.

Isolate number	Assigned accession number	Sequence length	Description	Accession number	% similarity	*E* value
BA1	KM882602	1205	*Stenotrophomonas maltophilia*	KJ584896	100	0.0
BA2	KM882603	1742	*Chryseobacterium* sp	KF640081	99	0.0
BA3	KM882604	1609	*Pseudomonas alcaligenes*	JX867714	99	0.0
BA4	KM882605	1777	*P. plecoglossicida*	JQ659971	100	0.0
BA5	KM882606	1687	*P. hibiscicola*	KC172017	99	0.0

## Data Availability

The sequences for each isolate have been deposited in the NCBI database under the accession numbers KM882602 (*S. maltophilia*), KM882603 (*Chryseobacterium* sp), KM882604 (*P. alcaligenes*), KM882605 (*P. plecoglossicida*), and KM882606 (*P. hibiscicola*).
